# Biliary Tract Carcinoma: Real-World Evidence in a Developing Country

**DOI:** 10.7759/cureus.108316

**Published:** 2026-05-05

**Authors:** Mauricio Lema, Camila Lema, Beatriz Preciado, Sergio Hoyos Duque, Mauricio Luján, Danilo Aguirre, Carlos Bonilla, Diego Gómez, Emilio Pérez, Mateo Pineda, Sara Patiño

**Affiliations:** 1 Oncology, Clínica de Oncologia Astorga, Medellín, COL; 2 Research Unit, Clínica de Oncología Astorga, Medellín, COL; 3 Gastrohepatology Research Group, Universidad de Antioquia, Medellín, COL; 4 Hepatobiliary (HPB) Unit and Liver Transplant Program, Hospital Pablo Tobon Uribe, Medellín, COL; 5 Oncology, Clínica de Oncología Astorga, Medellín, COL; 6 Oncology, Medicáncer, Medellín, COL; 7 Oncology, Sociedad de Oncología y Hematología del Cesar SAS (SOHEC), Valledupar, COL; 8 Department of Gastrointestinal and Neuroendocrine Tumors, Centro de Tratamiento e Investigación Sobre Cáncer Luis Carlos Sarmiento Angulo (CTIC), Bogotá, COL; 9 Oncology, Hospital Internacional de Colombia, Bucaramanga, COL; 10 Surgery, Universidad Pontificia Bolivariana, Medellín, COL

**Keywords:** biliary tract neoplasms, cholangiocarcinoma, gemcitabine, neoplasm metastasis, progression-free survival, survival analysis

## Abstract

Background

Biliary tract carcinoma (BTC) is underreported. This study describes the clinical characteristics and survival outcomes of BTC patients at six institutions in Colombia.

Methods

This is an observational, retrospective, multicenter, and real-life study of BTC patients. Baseline demographics, clinical characteristics, and treatment patterns were collected. A Kaplan-Meier analysis was used to estimate overall survival (OS), event-free survival (EFS), and progression-free survival (PFS). Multivariable Cox proportional hazards regression analyses for OS were performed in the overall cohort and in patients who received first-line treatment.

Results

A total of 214 BTC patients were included of which 136 were women (63.6%). Gallbladder carcinoma accounted for n=102 (47.7%), extrahepatic cholangiocarcinoma for n=59 (27.5%), and intrahepatic cholangiocarcinoma for n=53 (24.8%). Radical surgery was performed in 127 (59.3%) patients, 34 (26.8%) of whom remained relapse-free. First-line treatment for advanced disease was given to 116 patients (54.2%). Gemcitabine alone or in combination was used in 102 (87.9%) patients. The median EFS and OS were 16.4 months (95%CI: 12.3-20.6) and 21.5 months (95%CI: 14.2-28.8), respectively. Among patients initially diagnosed with metastatic disease, the median OS was 7.9 months (95%CI: 6.9-8.9). In 55 patients treated with gemcitabine-cisplatin as first-line therapy, the median OS was 9.6 months (95%CI: 5.5-13.7). In multivariable analysis, Eastern Cooperative Oncology Group performance status ≥2 and CA 19-9 >100U/mL were independently associated with worse OS.

Conclusions

In Colombia, patients with BTC face a poor prognosis, even in the early stages. Survival with gemcitabine-cisplatin for advanced disease is lower than that reported in clinical trials. These real-world data inform expectations for physicians and patients in Colombia.

## Introduction

Biliary tract carcinoma (BTC) comprises a heterogeneous group of infrequent, aggressive gastrointestinal malignancies, including cholangiocarcinoma (CCA) and gallbladder carcinoma (GBC) [1]. CCA is a group of epithelial tumors arising from the biliary tree, and it is the second most common cause of primary liver tumors after hepatocellular carcinoma, accounting for approximately 10-15% of primary liver cancers and 3% of all metastatic cancers [1,2]. CCA is classified according to its anatomical location as intrahepatic (iCCA) or extrahepatic cholangiocarcinoma (eCCA). The incidence of BTC has been increasing in recent decades. However, there are barriers to adequate classification owing to the inclusion of BTC with other primary liver malignancies in many tumor registries. For example, GLOBOCAN only includes gallbladder tumors, which typically represent 20-30% of BTCs [3].

Surgery followed by the adjuvant capecitabine is recommended for nonmetastatic BTC [4]. Cisplatin-based chemotherapy with durvalumab, an immune checkpoint inhibitor, improved overall survival (OS) to a median of 12.8 months and became the standard of care (TOPAZ-1) [5]. In the KEYNOTE-966, the addition of pembrolizumab to chemotherapy also improved OS in advanced BTC [6,7]. As a result of these trials, immune checkpoint inhibitors have become the standard of care in many countries, including the USA, the European Union, and Colombia. Targeted therapy may be an option for advanced BTC with proven oncogenic drivers for patients who have access to these agents.

Data on the epidemiology and outcomes of BTC in Colombia are limited. For 2022, GLOBOCAN estimates 669 GBC cases and 519 deaths, ranking 24th and 19th in frequency and mortality from cancer in the country, respectively [8]. To fill this void, this study describes the clinical characteristics and survival outcomes of patients diagnosed with BTC treated at different institutions in Colombia, including subgroup descriptions according to disease stage, treatment patterns, and tumor location.

The results of this study were previously presented as a meeting abstract and poster at American Society of Clinical Oncology (ASCO) GI 2026 on January 09, 2026.

## Materials and methods

Study design

This multicenter, retrospective, longitudinal, observational study was conducted in six medical centers. Participating institutions included academic centers and hospitals with oncology departments capable of providing comprehensive cancer care (Clínica de Oncología Astorga, Hospital Pablo Tobón Uribe [HPTU], and Medicáncer in Medellín; Sociedad de Hematología y Oncología del Cesar [SOHEC] in Valledupar; Centro de Tratamiento e Investigación sobre Cáncer Luis Carlos Sarmiento Angulo [CTIC] in Bogotá; and Hospital Internacional de Colombia [HIC] in Bucaramanga). The study was conducted between January and December 2024. For this study, the European Society for Medical Oncology (ESMO) Guidance for Reporting Oncology real-World Evidence (GROW) criteria were applied [9].

Patient selection

Patients were retrospectively identified from institutional medical records at each participating center. Consecutive patients who met the predefined eligibility criteria during the study period were included. Inclusion criteria comprised patients with a diagnosis of BTC including iCCA, eCCA, and GBC. Diagnoses were identified using the following International Classification of Diseases, 10th Revision (ICD-10) codes: C22.1, C23.x, C24.0, and C24.9. Additionally, patients had received surgical and/or systemic treatment at participating institutions. Patients whose clinical records were incomplete in relation to stage at diagnosis and/or treatment data were excluded.

Data collection

Clinical data collection included demographic information, tumor characteristics, data on diagnosis, therapeutic interventions, and survival outcomes. Information was collected retrospectively from patients’ medical records and entered into an electronic case report form (eCRF).

To ensure data quality, each participating institution conducted internal quality checks, validating the accuracy and completeness of the recorded information. Additionally, the staff of Clínica de Oncología Astorga (named the Central Team) periodically monitored the study sites. This monitoring included verifying the absence of duplicate enrolled subjects and ensuring adherence to the data collection protocol to safeguard data integrity.

Outcomes

The primary outcomes were event-free survival (EFS), progression-free survival (PFS), and overall survival 1 (OS1). EFS was measured from the date of treatment initiation to the date of recurrence/progression or death from any cause in patients classified as stage I to IV (M0). PFS was defined as the time from the start of treatment until recurrence/progression or death from any cause in patients with metastatic disease. OS1 is the time from BTC diagnosis to the last patient follow-up or death from any cause. Overall survival 2 (OS2) is a secondary outcome, defined as the time from the beginning of therapy for advanced disease to the last patient follow-up or death from any cause. Patients who were alive or free from progression/recurrence at the end of the study period or last follow-up were censored in the analyses of OS, PFS, and EFS, respectively.

Although this is not an experimental trial, we consider a median survival of at least 8.96 months to be acceptable for metastatic disease in this unselected, real-world patient population. This target represents 80% of the median survival observed in the gemcitabine and cisplatin arms of the ABC02 trial [4].

Statistical analysis

No formal sample size calculations were conducted. All patients who met the eligibility criteria were included.

The data was analyzed via SPSS version 22 (IBM Corp., Armonk, NY, USA). The Kolmogorov-Smirnov test was used to assess the normality of the quantitative variables. The median with interquartile range (IQR) was used to describe non-normally distributed data. Categorical variables are expressed with absolute and relative frequencies. Missing data were reported as unknown.

The non-parametric Kaplan-Meier method was used to estimate EFS, PFS, and OS curves for the overall cohort and stratified by stage at diagnosis. Additionally, clinically relevant subgroups were defined a priori based on investigator interest to further describe OS as part of the descriptive analysis. These subgroups were defined according to tumor anatomical site, treatment patterns, patients with stage IV BTC who underwent surgery to the primary tumor, patients initially diagnosed with metastatic disease, and patients who received gemcitabine plus cisplatin as first-line therapy. Differences were evaluated using the log-rank test. A p-value <0.05 was considered to indicate statistical significance.

A multivariable Cox proportional hazards regression analysis was performed in the overall cohort to evaluate factors independently associated with OS, regardless of their statistical significance in the univariable analysis. Covariates were selected a priori based on clinical relevance and biological plausibility, irrespective of their significance in univariable analyses, to account for potential confounding. The model included age at diagnosis, sex, Eastern Cooperative Oncology Group performance status (ECOG-PS), primary tumor site, and CA 19-9 levels. For this analysis, CA 19-9 was recategorized as ≤37 U/mL, 38-100 U/mL, and >100 U/mL, using 37 U/mL as the upper limit of normal [10]. Additionally, ECOG-PS was recategorized into two groups: 0-1 and 2-3. Hazard ratios (HR) and 95% confidence intervals (95%CI) were calculated to estimate the risk of death.

Additionally, a multivariable Cox regression analysis was performed in the subgroup of patients who received first-line treatment. Covariates were selected according to the same predefined clinical criteria. Disease presentation status (de novo vs recurrent) was not retained in the final model because sparse data within the poor performance status subgroup resulted in unstable parameter estimates.

## Results

**Figure 1:**
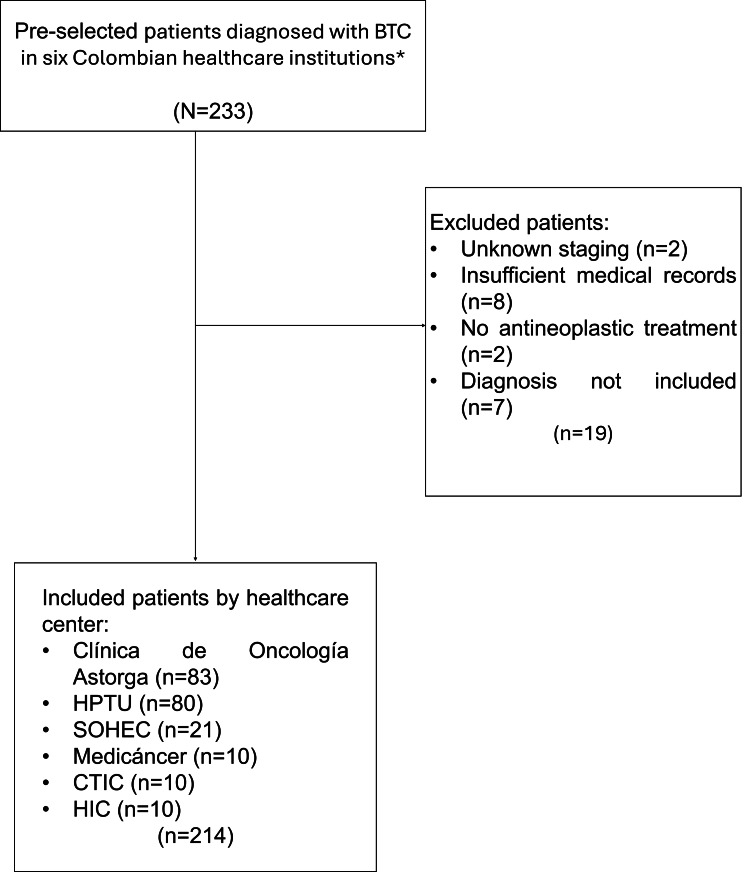
Flowchart presenting the selection of patients for data analysis. BTC: Biliary tract carcinoma. SOHEC: Sociedad de Hematología y Oncología del Cesar. CTIC: Centro de Tratamiento e Investigación sobre Cáncer. HIC: Hospital Internacional de Colombia. HPTU: Hospital Pablo Tobón Uribe.

Data from the 214 patients who met the eligibility criteria was analyzed (Figure 1).

The data collection cutoff was November 2024. The median age at diagnosis was 64.0 years (IQR: 57.0-71.0). Women represented 63.6% of the population (n=136). With respect to healthcare regime distribution, 50.0% belonged to the Contributive Regime (n=107) (Table 1). The primary site was GBC in 47.7% (n=102), eCCA in 27.6% (n=59), and iCCA in 24.8% (n=53) of the population. Of the 27.6% with eCCA (n=59), perihilar cholangicarcinoma and distal CCA were 35.6% (n=21/59) and 61.0% (n=36/59), respectively. No information was found regarding the location (distal or perihilar) in two patients diagnosed with eCCA.

**Table 1:** Sociodemographic and clinical characteristics of patients with biliary tract carcinoma. GBC: gallbladder carcinoma; iCCA: Inthahepatic cholangiocarcinoma; eCCA: Exthahepatic cholangiocarcinoma; Mdn (IQR): Median-Interquartile Range; ECOG-PS: Eastern Cooperative Oncology Group performance status. *No information was found regarding the location (distal or perihilar) in two patients diagnosed with extrahepatic cholangiocarcinoma.

Characteristics		GBC (n=102)		iCCA (n=53)		eCCA - Perihiliar (n=21)		eCCA- Distal (n=36)		eCCA- Total (n=59)		Overall cohort (N=214)	
		n	%	n	%	n	%	n	%	n	%	n	%
Age at diagnosis (years) – Mdn (IQR)		64 (58.0-71.0)		64 (57.0 - 72.5)		63 (53.5 - 69.0)		61.5 (53.0 - 71.0)		62 (53.0 - 71.0)		64 (57.0 - 71.0)	
Sex	Women	77	75.5	32	60.4	9	42.9	17	47.2	27	54.2	136	63.6
	Men	25	24.5	21	39.6	12	57.1	19	52.8	32	45.8	78	36.4
Healthcare System Affiliation	Contributive Regime	51	50	25	47.2	14	66.7	16	44.4	31	52.5	107	50
	Subsidized Regime	30	29.4	9	17	6	28.6	11	30.6	17	28.8	56	26.2
	Private health insurance	13	12.7	14	26.4	1	4.8	8	22.2	10	16.9	37	17.3
	Special Regimens	4	3.9	3	5.7	0	0	1	2.8	1	1.7	8	3.7
	Other	4	3.9	2	3.8	0	0	0	0	0	0	6	2.8
Histology	Adenocarcinoma	96	94.1	51	96.2	20	95.2	36	100	58	98.3	205	95.8
	Other	2	2	0	0	0	0	0	0	0	0	2	0.9
	Unknown	4	3.9	2	3.8	1	4.8	0	0	1	1.7	7	3.3
Tumor differentiation	Well-differentiated	25	24.5	12	22.6	9	42.9	17	47.2	26	44.1	63	29.4
	Moderately-differentiated	33	32.4	20	37.7	4	19	13	36.1	17	28.8	70	32.7
	Poorly-differentiated	17	16.7	5	9.4	3	14.3	5	13.9	8	13.6	30	14
	Unknown	27	26.5	16	30.2	5	23.8	1	2.8	8	13.6	51	23.8
Biomarkers	CA 19-9 - Mdn (IQR)	19.9 (6.16-142.0)		24.3 (13.8 - 367.0)		24.2 (12.2 - 390.2)		45.3 (12.0 - 278.0)		29.1 (12.1 - 278.0)		22.2 (11.5 - 196.6)	
	CEA - Mdn (IQR)	3.2 (1.8 - 26.3)		3 (1.8 - 7.5)		2.9 (1.4 - 3.3)		2 (1.7 - 4.4)		2.1 (1.7 - 4.0)		2.9 (1.7 - 8.3)	
Stage at diagnosis	I	4	3.9	14	26.4	6	28.6	12	33.3	18	30.5	36	16.8
	II	17	16.7	9	17	6	28.6	16	44.4	23	39	49	22.9
	III	40	39.2	4	7.5	4	19	5	13.9	9	15.3	53	24.8
	IV	41	40.2	26	49.1	5	23.8	3	8.3	9	15.3	76	35.5
	IVM0	5	12.2	2	7.7	0	0	0	0	0	0	7	9.2
	IVM1	36	87.8	24	92.3	5	100	3	100	9	100	69	90.8
ECOG-PS at diagnosis	0	31	30.4	15	28.3	6	28.6	10	27.8	17	28.8	63	29.4
	1	54	52.9	25	47.2	10	47.6	25	69.4	36	61	115	53.7
	2	3	2.9	2	3.8	1	4.8	1	2.8	2	3.4	7	3.3
	3	1	1	0	0	0	0	0	0	0	0	1	0.5
	4	0	0	0	0	0	0	0	0	0	0	0	0
	Unknown	13	12.7	11	20.8	4	19	0	0	4	6.8	28	13.1

The stage distribution was as follows: 16.8% for stage I (n=36), 22.9% for stage II (n=49), 24.8% for stage III (n=53), 3.3% for non-metastatic stage IV (M0) (n=7) and 32.2% for metastatic stage IV (M1) (n=69). The median CA 19-9 and CEA levels were 22.2 U/ml (IQR 11.5-196.6) and 2.9 ng/ml (IQR 1.7-8.3), respectively. For patients with available ECOG-PS data, 83.2% were scored 0 or 1 at the time of diagnosis (n=178) (Table 1).

With respect to the natural history of the disease of the different cancer sub-types, patients diagnosed with eCCA presented in earlier stages of the disease, so that approximately 40.0% of patients (n=23/59) were in stage II at the time of diagnosis (Table 1).

Next-generation sequencing (NGS) results were available for four patients (1.9%), revealing a pathogenic IDH gene mutation in two patients (1.0%), high microsatellite instability in one patient (0.5%), and Cyclin loss in one patient (0.5%). No patient received targeted therapy. The patient with high microsatellite instability was treated with pembrolizumab as a first-line treatment.

Treatment description

Surgery

Radical surgery was performed on 127 patients. Of these, 123 were among the 138 patients diagnosed with stage I-III disease at the time of diagnosis. Additionally, four patients diagnosed with stage IV (M0) GBC also underwent radical surgery (Table 2).

**Table 2:** Initial treatment description according to clinical stage. *Non-radical surgery in stage IV: All patients underwent surgery with the intent of performing radical resection, without prior staging indicating stage IV disease. Those classified as stage IV were identified either intraoperatively due to undetected implants or postoperatively through pathological analysis of the liver specimen in gallbladder surgery. ꝉThis is the first treatment given to patients after diagnosis.  ‡Fluoropyrimidine: 5- fluorouracil or capecitabine. §Platinum-based agents include cisplatin, carboplatin, and oxaliplatin.

Treatment Patterns Based on Stage at Diagnosis	I		II		III		IV(M0)		IV(M1)		Overall cohort	
	(n=36)		(n=49)		(n=53)		(n=7)		(n=69)		(n=214)	
	n	%	n	%	n	%	n	%	n	%	n	%
Patients who underwent surgery:	35	97.2	47	95.9	48	90.6	5	71.4	19	27.5	154	72
Intention of surgery	(n=35)		(n=47)		(n=48)		(n=5)		(n=19)		(n=154)	
Radical	35	100	44	93.6	44	91.7	4	80	0	0	127	82.5
Non-radical	0	0	3	6.4	4	8.3	1	20	19*	100	27	17.5
First chemotherapy regimen^ ꝉ^	19	52.8	28	57.1	35	66	5	71.4	68	98.6	155	72.4
Gemcitabine	4	21.1	7	25	2	5.7	0	0	3	4.4	16	10.3
Gemcitabine + Fluoropyrimidine^‡^	3	15.8	1	3.6	4	11.4	0	0	4	5.9	12	7.7
Gemcitabine + Cisplatin + Durvalumab	0	0	1	3.6	1	2.9	0	0	5	7.4	7	4.5
Gemcitabine + Platinum-based agents^§^	1	5.3	6	21.4	10	28.6	4	80	55	80.9	76	49
Gemcitabine + Nab-paclitaxel	0	0	0	0	0	0	0	0	1	1.5	1	0.6
Fluoropyrimidine	9	47.4	7	25	9	25.7	1	20	0	0	26	16.8
Fluoropyrimidine + Oxaliplatin	0	0	0	0	1	2.9	0	0	0	0	1	0.6
Paclitaxel + Carboplatin	0	0	0	0	1	2.9	0	0	0	0	1	0.6
Unknown	2	10.5	6	21.4	7	20	0	0	0	0	15	9.7
First chemotherapy scenario	(n=19)		(n=28)		(n=35)		(n=5)		(n=68)		(n=155)	
Neoadjuvant therapy	1	5.3	1	3.6	3	8.6	0	0	0	0	5	3.2
Adjuvant therapy	18	94.7	26	92.9	26	74.3	1	20	0	0	71	45.8
First-line therapy	0	0	1	3.6	6	17.1	4	80	68	100	79	51

Adjuvant or Neoadjuvant Therapy

Neoadjuvant protocols were given to five patients (2.3%), whereas adjuvant systemic regimens were administered to 71 patients (55.9% of patients who underwent radical surgery) (Table 2). Adjuvant regimens included fluoropyrimidine-based treatments in 26 patients (36.6%), single-agent gemcitabine in 13 patients (18.3%), and a combination of both in eight patients (11.3%).

Recurrence After Radical Surgery

**Figure 2:**
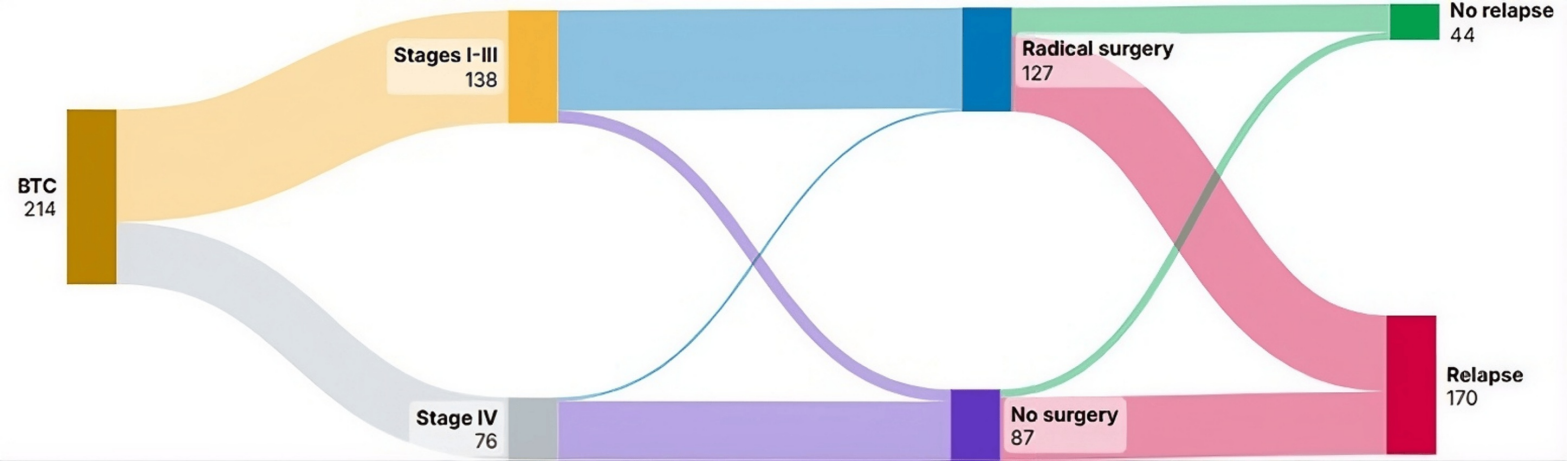
Diagram of the evolution of patients diagnosed with biliary tract carcinoma (BTC) until relapse or disease progression. *Patients diagnosed with stage IV (M0) gallbladder carcinoma. Image credits: Author. The Sankey diagram was created by the authors using the Sankey Art platform (https://www.sankeyart.com).

Recurrence was documented in 62 out of 123 patients (50.4%) with stage I-III disease who underwent radical surgery. Similarly, recurrence occurred in three out of four GBC patients (75.0%) with stage IV (M0) disease who underwent radical surgery (Figure 2).

First-Line Treatment

A total of 116 patients (54.2%) received first-line systemic treatment for advanced disease. Among these patients, 73 patients (62.9%) were classified as having advanced disease at the beginning of treatment, while 38 patients (32.8%) had previously undergone surgery, including 35 with radical surgery (Table 2).

The most common first-line systemic treatment for advanced disease was gemcitabine, which was used either alone or with other drugs, in 102 patients (87.9%). Among these, 77 patients received a combination of gemcitabine and platinum, and eight patients received gemcitabine, platinum, or durvalumab. Additionally, 14 patients were treated with fluoropyrimidine-based regimens, either alone or in combination with other agents.

Second-Line Treatment

Among the patients who received first-line treatment, 25 (21.6%) transitioned to second-line therapy. The most frequently used regimens were fluoropyrimidine-based and were administered either as monotherapy or in combination in 22 patients (88.0%).

Treatment response

Treatment response was only evaluated for patients who were given systemic treatment initially in a neoadjuvant scenario (n=5) or as a first-line treatment for advanced disease (n=76). Among these patients, treatment response was documented in 58 cases (Table 3). The median time between the initiation of treatment and response assessment was 15.6 weeks (IQR 12.0 - 22.0).

**Table 3:** Treatment response among evaluable patients.

Treatment response	Evaluable patients (n=58)	
	n	%
Complete response	2	3.4
Stable disease	21	36.2
Partial response	9	15.5
Progressive disease	26	44.8

Time-to-event outcomes

The clinical outcomes associated with the different treatments were evaluated after a median follow-up time of 16.7 months (IQR 9.1-47.3). At the end of follow-up, 61 patients (28.5%) were alive.

**Figure 3:**
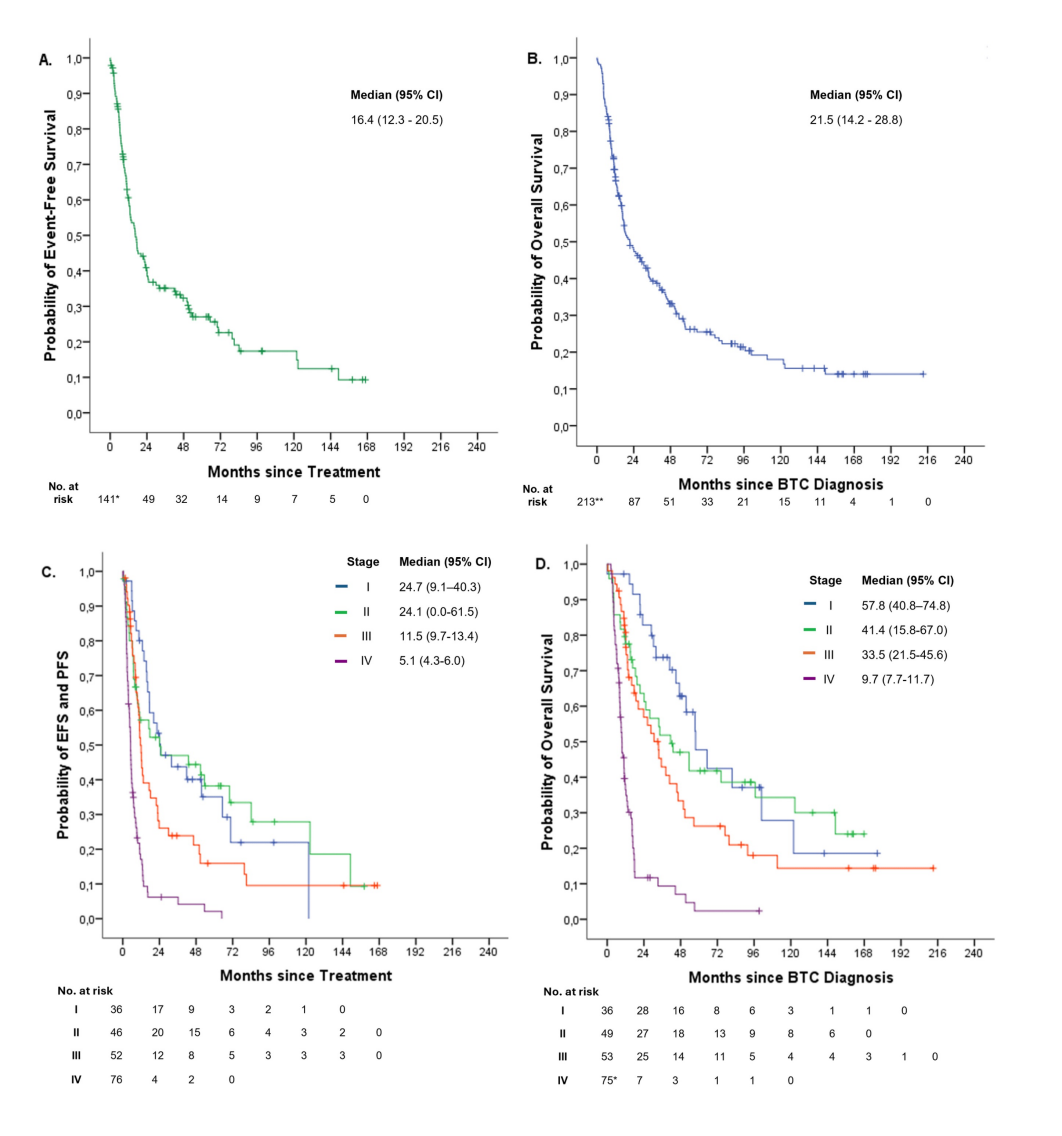
Kaplan-Meier curves for: (A) event-free survival, (B) overall survival for the entire cohort, (C) event-free survival (EFS) or progression-free survival (PFS), and (D) overall survival by disease stage in patients with biliary tract carcinoma (BTC). No. at risk indicates the number of patients at risk at each time point. *Data from 141 patients (progression date could not be defined in four patients) were included in the event-free survival analysis. **Overall survival analysis was performed with information from 213 patients (the date of diagnosis could not be determined for one patient).

For the entire cohort, the median EFS (mEFS) was 16.4 months (95% CI: 12.3-20.5) (Figure 3A), and the median OS (mOS) was 21.5 months (95% CI: 14.2-28.8) (Figure 3B). 

Outcomes were analyzed by disease stage subgroup. The mEFS for stages I, II, and III disease was 24.7 months (95% CI: 9.1-40.3), 24.1 months (95% CI: 0.0-61.5) and 11.5 months (95% CI: 9.7-13.4), respectively. The median PFS for stage IV patients was 5.1 months (95% CI: 4.3-6.0). The differences observed between stages were statistically significant (p < 0.01) (Figure 3C).

With respect to OS1, stage I patients had a mOS of 57.8 months (95% CI: 40.8-74.8), 41.4 months (95% CI: 15.8-67.0) for stage II patients, 33.5 months (95% CI: 21.5-45.6) for stage III patients, and 9.7 months (95% CI: 7.7-11.7) for stage IV patients (Figure 3D). The differences observed between groups were statistically significant (p < 0.01). Surgery to the primary site was performed in 24 out of 75 patients with stage IV BTC. Median survival was 12.8 months (95% CI: 6.7-18.9 months) in the operated group and 9.5 months (95% CI: 7.6-11.3 months) in the non-operated group. This difference did not reach statistical significance (p = 0.19).

**Figure 4:**
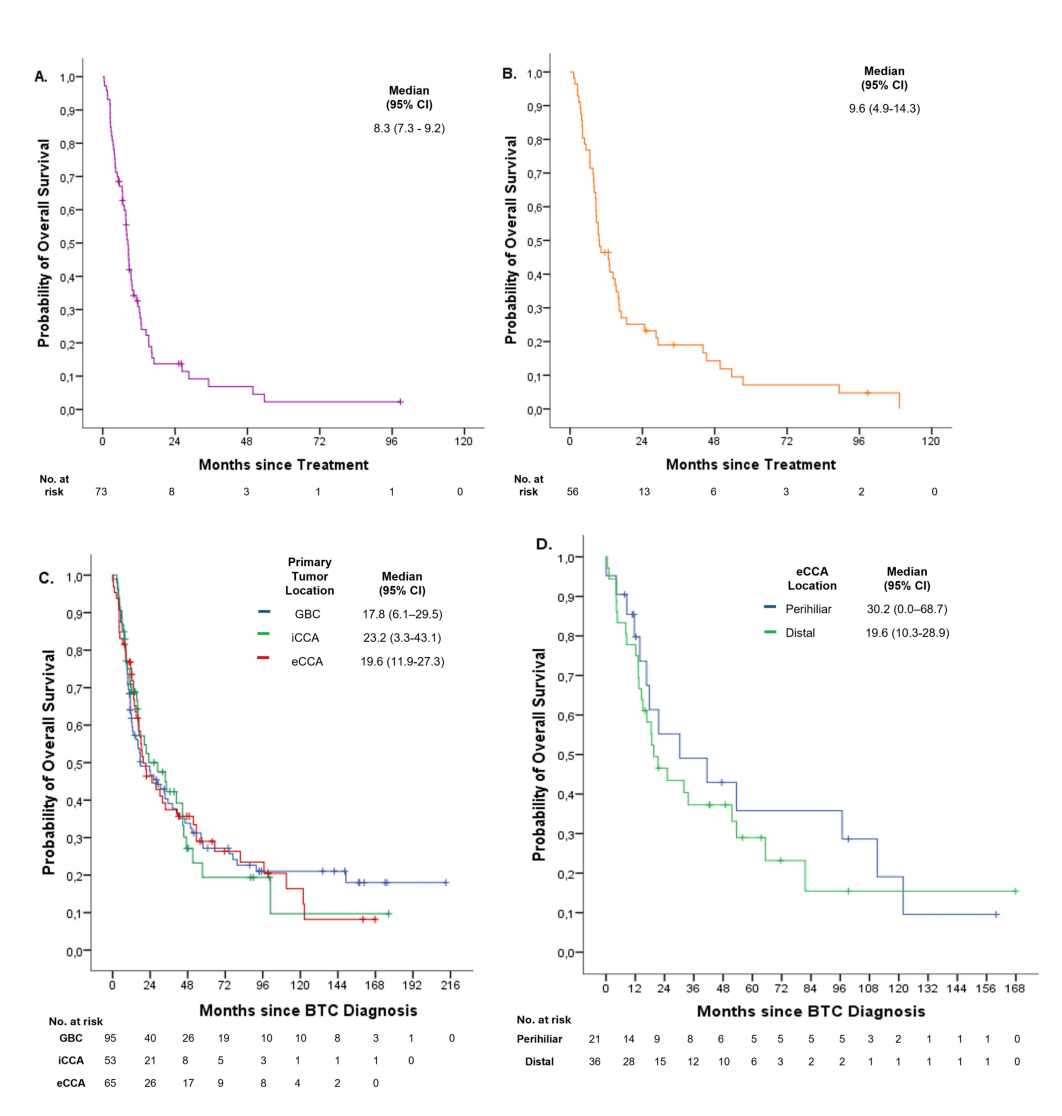
Kaplan-Meier curves for overall survival for: (A) de novo metastatic biliary tract carcinoma (BTC) patients, (B) patients treated with gemcitabine plus cisplatin as first-line therapy, (C) patients according to primary tumor location, and (D) patients according to extrahepatic cholangiocarcinoma location. No. at risk indicates the number of patients at risk at each time point. GBC: gallbladder carcinoma. iCCA: intrahepatic cholangiocarcinoma. eCCA: extrahepatic cholangiocarcinoma.

The mOS2 in the subgroup of patients who were initially diagnosed with metastatic disease (n=69) was 7.9 months (95% CI: 6.9-8.9) (Figure 4A). Notably, the mOS2 for the 55 patients treated with gemcitabine plus cisplatin as first-line therapy was 9.6 months (95% CI: 5.5-13.7) (Figure 4B). 

The mOS1 values according to primary tumor location were as follows: 17.8 months (95% CI: 6.1-29.5), 19.6 months (95% CI: 11.9-27.3), and 23.2 months (95% CI: 3.3-43.1) for GBC, eCCA, and iCCA, respectively (p=0.99) (Figure 4C). The mOS1 for eCCA was additionally stratified by anatomical location: patients with perihilar tumors had an mOS1 of 30.2 months (95% CI: 0.0-68.7), while those with distal tumors had an mOS1 of 19.6 months (95% CI: 10.3-28.9) (Figure 4D).

Prognostic factors

In the multivariable Cox regression analysis of the overall cohort, ECOG-PS 2-3 (HR 3.83, 95% CI 1.55-9.44; p=0.004) and CA 19-9 >100 U/mL (HR 2.39, 95% CI 1.42-4.01; p=0.001) were independently associated with worse OS. Age, sex, and primary tumor site were not significantly associated with OS. Both ECOG-PS and CA 19-9 were also predictive of worse OS in the subgroup of patients who received first-line treatment (Table 4).

**Table 4:** Univariable and multivariable Cox proportional hazards regression analysis for overall survival in patients who received first-line treatment HR: hazard ratio; CI: confidence interval; ECOG-PS: Eastern Cooperative Oncology Group performance status; GBC: gallbladder cancer; iCCA: intrahepatic cholangiocarcinoma; eCCA: extrahepatic cholangiocarcinoma.

Variables	Category	Univariable HR (95% CI)	p-value	Multivariable HR (95% CI)	p-value
Age at diagnosis	NA	1.01 (0.99-1.02)	0.57	0.97 (0.94-1.00)	0.07
Sex	Women	Reference	—	Reference	—
	Men	1.18 (0.78-1.79)	0.44	0.98 (0.47-2.03)	0.96
ECOG-PS	0-1	Reference	—	Reference	—
	2-3	2.53 (1.07-5.98)	0.04	3.37 (1.22-9.32)	0.02
Primary site tumor	GBC	Reference	—	Reference	—
	iCCA	0.61 (0.38-0.97)	0.04	0.64 (0.30-1.40)	0.27
	eCCA	0.84 (0.49-1.43)	0.52	0.79 (0.32-1.95)	0.61
CA 19-9 (U/mL)	≤37	Reference	—	Reference	—
	38-100	0.81 (0.30-2.16)	0.67	0.62 (0.17-2.24)	0.47
	>100	2.07 (1.14-3.73)	0.02	2.41 (1.17-4.95)	0.02
Metastatic disease	de novo	Reference	—	Reference	—
	Recurrence	1.12 (0.58-2.14)	0.74		

## Discussion

The primary objective of this study was to determine the demographic and clinical characteristics of patients diagnosed with BTC in several oncologic centers in Colombia. The secondary objectives included characterizing treatment patterns associated with the management of BTC and time-to-event metrics for EFS, PFS and OS.

In summary, among our cohort of 214 patients, there was a predominance of females, accounting for 63.6% (n=136), corresponding to a female-to-male ratio of 1.74:1. This finding is consistent with the documented higher incidence of gallbladder cancer in women, where a female predominance of approximately 2:1 has been reported in the literature [11]. In contrast, the European BTC population has a male predominance of 62.6% [12]. Gallbladder cancer was also the most common type observed, representing nearly half of the cases. This aligns with data from the Surveillance, Epidemiology, and End Results (SEER) program, which reports that gallbladder cancer accounts for 41.3% of biliary tract cancers in the United States [13]. The majority of patients had early-stage BTC (stage I-III) at initial diagnosis, and approximately half progressed to advanced disease, while one-third had late-stage/advanced BTC (stage IV) at initial diagnosis, and the vast majority of patients had a performance status of 0 or 1. Radical surgery was performed in nearly 90% of patients with stage I-III disease. However, approximately half of these patients experienced recurrence during follow-up. Adjuvant or neoadjuvant chemotherapy was administered to approximately two-thirds of the patients who underwent surgery. After a median follow-up of more than 21 months, nearly 40% of stage I/II/III patients are still alive. Patients with stage IV (M0) disease who underwent radical surgery had a very high recurrence rate.

In our cohort, 55% of patients received first-line chemotherapy for advanced disease. Among these patients, two-thirds presented with de novo metastatic disease, while the remaining patients relapsed after treatment with curative intent. The predominant first-line chemotherapy regimens were gemcitabine plus platinum-based agents, which is consistent with current standards of care for advanced BTC [14,15]. The use of durvalumab was limited to a minority of patients, as this agent was only approved in Colombia in 2024. In our study population, the response rate was 18%, highlighting the aggressive nature of these malignancies. Among patients with metastatic BTC, the median PFS was 5.1 months, and the mOS was 7.9 months; these results appear inferior to those reported in clinical trials for advanced disease, which may be explained by the inclusion of real-world patients with more heterogeneous clinical characteristics [4,5]. Only one-fifth of the patients who initiated first-line therapy received second-line therapy. Second-line chemotherapy was mostly fluoropyrimidine-based. After each line of therapy, there was a steep decrease in the number of patients receiving subsequent lines of therapy. Personalized medicine was not available for this cohort. The mOS1 was 57.8 months, 41.4 months, 33.5 months, and 9.7 months for stages I, II, III, and IV, respectively. No significant differences in mOS1 were observed among patients with GBC, iCCA, or eCCA. The median survival for distal eCCA was 19.6 months, compared to 30.2 months for perihilar eCCA. This numerical difference suggests a worse prognosis for distal eCCA, despite the fact that this group presented with earlier-stage disease. The significance of this finding remains uncertain, as widespread misclassification of the primary site in BTC has been reported in large cohorts [4,5,12,16].

One relevant finding of this study was the consistent prognostic impact of both ECOG-PS and CA 19-9 levels. Poor ECOG-PS was independently associated with worse OS in both the overall cohort and in the subgroup of patients who received first-line treatment, highlighting functional status as a key determinant of outcomes in BTC. Our findings are consistent with prior real-world data showing ECOG-PS as one of the strongest independent predictors of survival in advanced BTC [17]. This observation is clinically meaningful, as ECOG-PS remains a readily available and reproducible variable that may help guide treatment decisions and patient counseling in routine practice.

Similarly, CA 19-9 levels >100 U/mL were independently associated with worse OS in both the overall cohort and the first-line treatment subgroup, suggesting that elevated tumor marker levels may reflect greater disease burden or more aggressive tumor biology at presentation. These findings are consistent with a recent meta-analysis in CCA, as well as multiple retrospective series, supporting the role of CA 19-9 as a surrogate marker of tumor burden and adverse tumor biology [18].

Interestingly, age, sex, and primary tumor site were not independently associated with survival after adjustment. These results are in agreement with other real-world evidence datasets [19]. These findings suggest that functional status and biological markers may have greater prognostic relevance than demographic characteristics or anatomical tumor location in our real-world BTC population.

Although a recent publication has explored prognostic scoring systems for BTC [20], these models are not routinely used in Colombia and are difficult to validate retrospectively, given that many of their constituent variables are time-dependent and may fluctuate significantly during the disease course. Therefore, simple and reproducible variables such as ECOG-PS and CA 19-9 may be more applicable in resource-constrained real-world settings. In addition, a contemporary cohort of patients treated with cisplatin, gemcitabine, and durvalumab [21] did not demonstrate a clear prognostic impact of metastatic site or metastatic burden on OS. Together with our findings, this supports the concept that functional status and biological markers may be more relevant prognostic determinants than anatomic disease distribution in real-world BTC practice, particularly in resource-constrained settings.

To the best of our knowledge, this is the first study to document the clinical characteristics and survival outcomes of patients with BTC in Colombia. As a result, there is no prior data available for direct comparison. The ABC-02 trial reported an mOS of 11.7 months (95% CI: 9.5-14.3 months) for patients receiving first-line gemcitabine plus cisplatin [4]. Similarly, the TOPAZ-1 and KEYNOTE-966 trials, which evaluated immune checkpoint inhibitors in combination with gemcitabine and cisplatin versus a placebo with the same chemotherapy regimen, reported that patients in the placebo group had mOS of 11.5 months (95% CI: 10.1 - 12.5 months), 12.8 months (95% CI: 11.1-14.0) and 12.7 months (95% CI: 11.5 - 3.6) for durvalumab and pembrolizumab, respectively [5,6]. In contrast, our study revealed that patients with de novo stage IV disease had a mOS2 of 7.9 months (95% CI: 6.9-8.9). Among those treated with gemcitabine and cisplatin, the mOS2 was 9.6 months, exceeding our predefined futility boundary. However, this survival duration is at least two months shorter than that reported in the ABC-02 trial, raising concerns about the generalizability of phase III trial results to a real-world population in Colombia [22,23].

The longer survival outcomes observed in the ABC-02, TOPAZ-1, and KEYNOTE-966 trials are expected, as these studies enrolled patients who met strict inclusion and exclusion criteria, resulting in a highly selected patient population [4-7]. While these data confirm the superiority of the respective experimental arms in controlled trial settings, the true effectiveness of this treatment strategy can be fully assessed only in real-world scenarios, where patients often present with comorbidities and other factors that may negatively impact survival, such as access barriers that hinder access to medications and limit compliance, potential differences in response to and tolerability of anticancer agents that may be unique to a genetic and ethnic make-up in Colombia.

The European Society of Medical Oncology recommends NGS for patients with advanced BTC because of the high frequency of genomic alterations and the potential benefit of targeted agents in these tumors [24]. In this cohort, however, genomic data and targeted therapy were largely absent, with NGS performed in only 1.9% of cases. The lack of approval and reimbursement of targeted agents and barriers to NGS technology may explain these observations.

Data from 309 CCA patients were collected from five referral centers across Latin America in a real-world setting, none of which were in Colombia [25]. This study revealed ethnic disparities in survival outcomes, with African and Hispanic patients showing lower survival rates than Caucasian patients when treated with chemotherapy for advanced disease.

In our study, ethnic affiliations were not recorded; however, most Colombians can be classified as Hispanic. Notably, the mOS for advanced disease in our cohort closely aligns with that of the Hispanic subgroup in the Latin American study, both approximately eight months [25]. In this real-world evidence dataset, the mOS for advanced GBC patients ranged from 6.5 to eight months. In another cohort of 786 BTCs in Mexico, a female predominance was also observed in patients with GBC and iCCA, with women comprising 79% and 71% of the cases, respectively [26]. The mOS for advanced disease was reported as nine months for iCCA, 12 months for eCCA, and only one month for GBC. The authors did not provide an explanation for the notably short median survival in GBC within their cohort. In our study, the median survival for advanced GBC patients was 9.7 months, which was superior to the reported outcomes in both the Latin American and Mexican cohorts [25,26].

In contrast, real-world data from Europe show a different scenario. A study conducted in Italy reported a mOS of 13.1 months for advanced disease in a cohort of 120 patients. Similarly, a pooled analysis of 792 patients with advanced BTC from France, Germany, Italy, Spain, and the United Kingdom reported a mOS of 13.4 months [12,27]. Within this analysis, Germany had the highest OS at 18.8 months, whereas the United Kingdom had the lowest OS at 8.8 months. The authors attributed the poorer survival outcomes in the UK to an older patient population, worse performance status at presentation, and longer intervals between diagnosis and initiation of therapy. Notably, survival outcomes in the UK are comparable to those reported in our study.

These differences underscore how survival outcomes vary across different settings. Recognizing such disparities is the first step toward improving them. Both the da Fonseca study [25] and our Colombian cohort demonstrated consistent but suboptimal survival rates for patients with BTC. To improve outcomes for BTC patients in Colombia, there is an urgent need to expand access to high-quality surgical care, adjuvant therapy, appropriate molecular profiling, immunotherapy, and targeted treatments for advanced disease. Without addressing these factors, significant improvements in BTC outcomes are unlikely to occur.

The limitations of our study include its observational, retrospective design, which may have resulted in incomplete data at the time of collection. Additionally, real-world evidence studies are inherently limited to evaluating associations rather than establishing causality. The lack of randomization and blinding further increases the risk of bias and confounding [28]. Furthermore, the use of convenience sampling may limit the representativeness of the cohort, as the study population may not fully reflect the broader population of patients with BTC [29].

In addition to these limitations, several additional factors should be considered. The variability in treatment protocols and clinical practices across institutions and regions in Colombia may have contributed to the heterogeneity observed within our cohort. Furthermore, the rapidly evolving landscape of BTC management, particularly with the introduction of targeted therapies and immunotherapies, may not be fully captured in our findings due to the retrospective nature of the study and the limited availability of these treatments during part of the study period. Molecular profiling was also not consistently available for all patients, which may have limited the evaluation of biomarker-driven therapies. Finally, the treatment access barriers commonly encountered in middle-income countries may have influenced treatment patterns and outcomes in real-world settings.

Despite these challenges, our study provides valuable insights into the management of BTC in Colombia, emphasizing its aggressive nature, genetic diversity, and limited availability of targeted therapy options for patients in this cohort.

## Conclusions

In conclusion, this study seeks to address the scarcity of Colombian data on BTC, which has hindered a comprehensive understanding of disease trends and outcomes. This study provides important insights into the BTC landscape, treatment patterns, and patient outcomes in Colombia. ECOG-PS and CA 19-9 are prognostic factors for OS in this real-world evidence study. Notably, survival outcomes in Colombia are lower than those reported in the literature. This reality should be carefully considered by both physicians and patients managing BTC in Colombia.
